# Single motor unit firing rate after stroke is higher on the less-affected side during stable low-level voluntary contractions

**DOI:** 10.3389/fnhum.2014.00518

**Published:** 2014-07-17

**Authors:** Penelope A. McNulty, Gaven Lin, Catherine G. Doust

**Affiliations:** ^1^Neuroscience Research AustraliaSydney, NSW, Australia; ^2^School of Medical Sciences, UNSW AustraliaSydney, NSW, Australia

**Keywords:** hemiparesis, motor unit firing rate, motor unit firing variability, torque control, stroke

## Abstract

Muscle weakness is the most common outcome after stroke and a leading cause of adult-acquired motor disability. Single motor unit properties provide insight into the mechanisms of post-stroke motor impairment. Motor units on the more-affected side are reported to have lower peak firing rates, reduced discharge variability and a more compressed dynamic range than healthy subjects. The activity of 169 motor units was discriminated from surface electromyography in 28 stroke patients during sustained voluntary contractions 10% of maximal and compared to 110 units recorded in 16 healthy subjects. Motor units were recorded in three series: ankle dorsiflexion, wrist flexion and elbow flexion. Mean firing rates after stroke were significantly lower on the more-affected than the less-affected side (*p* < 0.001) with no differences between dominant and non-dominant sides for healthy subjects. When data were combined, firing rates on the less-affected side were significantly higher than those either on the more-affected side or healthy subjects (*p* < 0.001). Motor unit mean firing rate was higher in the upper-limb than the lower-limb (*p* < 0.05). The coefficient of variation of motor unit discharge rate was lower for motor units after stroke compared to controls for wrist flexion (*p* < 0.05) but not ankle dorsiflexion. However the dynamic range of motor units was compressed only for motor units on the more-affected side during wrist flexion. Our results show that the pathological change in motor unit firing rate occurs on the less-affected side after stroke and not the more-affected side as previously reported, and suggest that motor unit behavior recorded in a single muscle after stroke cannot be generalized to muscles acting on other joints even within the same limb. These data emphasize that the less-affected side does not provide a valid control for physiological studies on the more-affected side after stroke and that both sides should be compared to data from age- and sex-matched healthy subjects.

## Introduction

Stroke is the leading cause of adult-acquired motor disability in developed countries (WHO, 2003). The most common outcome after stroke, and the most common cause of motor disability, is hemiparesis or weakness on the side of the body contralateral to the stroke lesion (e.g., Chang et al., 2013). Although the acute lesion is restricted to the brain, secondary adaptive and maladaptive changes may contribute to hemiparesis. There are four principal sites where such degeneration has the capacity to contribute to muscle weakness: (i) cerebral diaschisis (Feeney and Baron, 1986); (ii) reduced corticospinal tract integrity (Fries et al., 1993; Pineiro et al., 2000; Sterr et al., 2010; Stinear et al., 2012); (iii) changes to peripheral motor axon properties (Jankelowitz et al., 2007; Huynh et al., 2013); and (iv) anatomical and physiological changes within the muscle and its constituent single motor units. This study will consider single motor unit discharge behavior.

There are both anatomical and physiological changes within the muscles of the more-affected side after stroke. The anatomical changes may include disuse atrophy (Jørgensen and Jacobsen, 2001; Ryan et al., 2002; Hara et al., 2004; Arasaki et al., 2006; Li et al., 2011); altered muscle phenotype (Jakobsson et al., 1991; De Deyne et al., 2004; Lukács et al., 2008; McKenzie et al., 2009); and reinnervation (Dattola et al., 1993; Hara et al., 2004; Lukács, 2005). Physiological changes include altered motoneuron pool activation so that there is a reduction in the mean motor unit discharge rate and the variability of this discharge (Rosenfalck and Andreassen, 1980; Dietz et al., 1986; Gemperline et al., 1995; Chou et al., 2013); disrupted recruitment threshold (including lower recruitment thresholds, reversed recruitment thresholds so that fast motor units are recruited before slower motor units, and a reduced range over which recruitment occurs), reduced modulation of firing rates, and compression of the dynamic range of motor unit discharge rates (Rosenfalck and Andreassen, 1980; Gemperline et al., 1995; Hu et al., 2012; Chou et al., 2013). Such changes contribute not only to hemiparesis, but also to reduced control of muscles on the more-affected side after stroke.

Single motor units are the smallest functional division of muscles. They represent the most distal component of the motor pathway and their discharge behavior reflects the intrinsic properties of both the motoneuron and the muscle fibers in addition to the net synaptic drive through this pathway. Recent data from our group recorded during post-stroke therapy demonstrated that the activity of isolated single motor units in severely paretic muscles precedes the development of compound muscle activity (i.e., multiple motor units recruited through voluntary commands), and that this progression is a hallmark of improved movement ability, even many years post-stroke (see McNulty et al., 2013; Thompson-Butel et al., 2013). To understand the process of recovery from isolated single motor unit activity to compound activity during dynamic movements it is simpler to begin with more controlled static tasks so that changes in the properties of single motor units, and the mechanisms controlling this behavior, can be investigated more systematically. The aim of this study was to examine the pattern of motor unit behavior during sustained static contractions.

The changes in motor unit discharge properties noted above have been measured over brief periods, usually from 5–20 s with a range of different tasks and levels of voluntary contraction. Each of these differences may be sufficient to alter the net synaptic drive to the motoneuron pool. For this reason, we extracted the action potentials of single motor units that were either spontaneously active or task-driven during a sustained isometric voluntary contraction at a functionally relevant duration and force intensity during ankle dorsiflexion, wrist flexion and elbow flexion. Motor units were recorded from both the more- and less-affected side after stroke and on both the dominant and non-dominant side in healthy subjects. Motor unit activity during contractions acting on three joints was studied because there are anatomical and functional differences in the control of muscles in the upper and lower limbs, and between proximal and distal muscles of the upper-limb. These differences include different innervation ratios (Buchthal and Schmalbruch, 1980), more numerous monosynaptic corticospinal (Palmer and Ashby, 1992) or bilateral (Colebatch et al., 1990) projections, and differences in mean firing rates (Petajan and Philip, 1969; de Luca, 1985). These differences are superimposed on functional recovery after stroke that is typically greater for the lower-limb than for the upper-limb although the reason for this is not clear. To ensure the results of this study do not simply reflect the differences listed here, data were collected during contractions at three joints. We compared differences in firing rates and the variability of the firing rate between sides and between the upper and lower limb. Data were recorded during elbow flexion from stroke subjects only to examine the effect of hand dominance on the control of motor unit behavior after stroke. Our results suggest that although motor units on the more-affected side have a reduced firing rate compared to the less-affected side as reported previously, the important difference is that the firing rate of motor units on the less-affected side after stroke is higher than both the more-affected side and motor units of healthy subjects.

## Materials and methods

### Subjects

**Figure 1:**
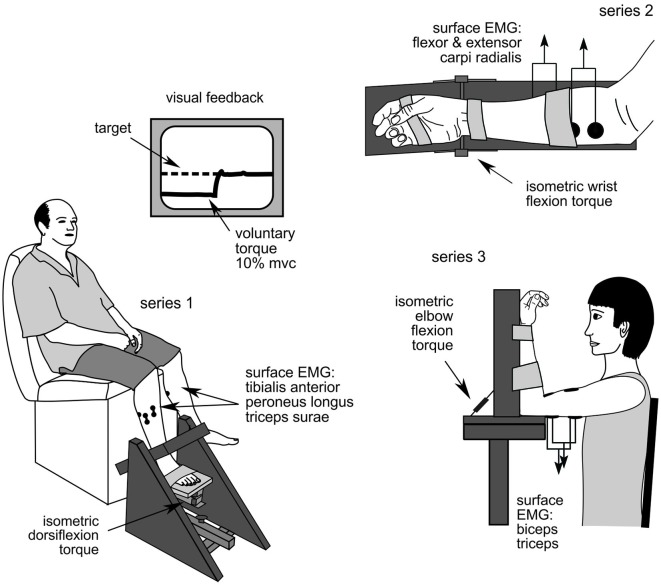
**Experimental set up**. Series 1: ankle dorsiflexion; series 2: wrist flexion; and series 3: elbow flexion.

The activity of 169 single motor units in 28 stroke patients was recorded in the course of three studies of low-level isometric force control: series 1: ankle dorsiflexion; series 2: wrist flexion; and series 3: elbow flexion (Figure 1). Activity from 110 single motor units was recorded in 16 healthy subjects who participated in the ankle and wrist experiments. Stroke patients were hemiparetic after a unilateral stroke with muscle weakness in the test limb. Those participating in series 1 could walk >15 m unassisted and none used lower-limb splints, braces or orthoses. Patients in series 2 and 3 had voluntary movement ≥10° at the test joint. Control subjects were neurologically healthy at the time of testing and all participants were cognitively competent (assessed as a Mini-Mental State Examination score ≥24). Participants were excluded if they had uncorrected vision or hearing, unstable blood pressure, or co-morbidities other than stroke that significantly affected sensorimotor function. Five patients and two healthy subjects participated in both the lower-limb and wrist experiments (participant demographics are presented in Table 1). All participants gave written, informed consent and these studies were approved by the Human Research Ethics Committees of the University of New South Wales and St Vincent’s Hospital, Sydney. Experiments were conducted in accordance with the Declaration of Helsinki.

**Table 1:** **Participant demographics and summary of single motor unit recordings**.

**Series**		**1 Ankle dorsiflexion**	**2 Wrist flexion**	**3 Elbow flexion**
Stroke patients	age (years; mean ± SD)	61.5 ± 11.6 (45–75)	65.3 ± 14.6 (42–83)	60.5 ± 16.4 (23–75)
	n (female, male)	10 (2, 8)	7 (2, 5)	11 (3, 8)
	time post-stroke (months)	59.0 ± 17.6 (7–168)	15.0 ± 4.8 (1–38)	31.4 ± 15.8 (3–150)
	walking speed (m.s^−1^)	1.1 ± 0.2 (0.4–2.1)		
	Fugl-Meyer upper-limb motor subscale (score)		42.1 ± 5.2 (14–57)	55.2 ± 2.6 (40–65)
	SMU count (units per patient, mean)	80 (8.0)	54 (8.5)	38 (4.8)
	SMU more-affected, less-affected side	45, 35	31, 23	21, 17
	SMU task driven, spontaneous	25, 55	15, 39	6, 32
	SMU ipsilateral (active side), contralateral to active side	45, 35	29, 25	21, 17
Healthy subjects	age (years; mean ± SD)	60.9 ± 11.6 (45–72)	55.7 ± 14.7 (39–71)	
	n (female, male)	10 (2, 8)	6 (2, 4)	
	SMU count (units per subject, mean)	67 (6.7)	43 (7.2)	
	SMU dominant, non-dominant side	23, 44	8, 35	
	SMU task driven, spontaneous	23, 44	9, 34	
	SMU ipsilateral (active side), contralateral to active side	15, 52	17, 26	

Note that the maximum upper-limb motor Fugl-Meyer Assessment score is 66. There is no control subject matched to the oldest stroke patient for the wrist flexion series (83 years), if this patient is excluded the stroke patients in series 2 were aged 62.3 ± 13.5 years (range 42–74 years). Data are presented as mean ± standard error of the mean (SEM) unless indicated otherwise. Note that motor units in series 1 were recorded from 4 EMG channels, and from 3 EMG channels in series 2 and 3. SMU: single motor unit.

### Experimental procedure

All data were recorded bilaterally with the exception of ankle dorsiflexion torque which was recorded on the active side only. Single motor unit electromyography (EMG) potentials were recorded using transducers (single motor unit (SMU) electrodes) specifically designed for this purpose with two parallel recording surfaces 1 × 10 mm, fixed 10 mm apart (DE2.3, Delsys, USA). The SMU electrode position was optimized prior to data collection and was not repositioned. Additional surface EMG data were recorded using standard 10 mm Ag/AgCl electrodes positioned in a belly-tendon montage with ~40 mm interelectrode distance (120 mm for triceps surae), hereafter referred to as EMG electrodes.

#### Series 1: ankle dorsiflexion

Participants sat with the knee in ~120° extension and the foot securely strapped to the myograph with the ankle positioned at the mid-point of passive range-of-motion (Figure 1). Torque was recorded on the test side only. The SMU electrodes were positioned over tibialis anterior with additional EMG electrodes over tibialis anterior, peroneus longus and triceps surae muscles.

#### Series 2: wrist flexion

Participants sat with the forearm and hand securely strapped to the myograph in a semi-pronated position with the wrist flexed to 30° (Figure 1). The digits were unconstrained. The SMU electrode was positioned over flexor carpi radialis muscle with additional EMG electrodes over flexor and extensor carpi radialis muscles.

#### Series 3: elbow flexion

Participants sat with the elbow supported by the myograph so that the shoulder and elbow were flexed to 90° and the forearm supinated and securely restrained (Figure 1) or as close as possible to this position for patients with joint stiffness or spasticity. The hand was unconstrained. The SMU electrode was positioned over biceps brachii muscle with EMG electrodes over biceps and triceps brachii muscles.

Single motor unit data were amplified 100–1000 times, bandpass filtered 20–450 Hz (custom amplifier) and digitized at 5 kHz. All other EMG data were amplified 200–1000 times, band-pass filtered 10–1,000 Hz (1902, CED, UK; or IP511, Grass, USA) and sampled at 5 kHz. Torque data were recorded using either a 1 kN (series 1–2) or 2 kN (series 3) load cell (Applied Measurements, Australia) amplified 175–550 times, filtered DC-20 Hz (2044B, Applied Measurements, Australia) and sampled at 2 kHz. All data were digitized and recorded using a 1401 data acquisition card and Spike2 software (CED, UK).

### Protocol

Maximum voluntary contraction (MVC) torque was measured on each side as the peak torque recorded during three brief (2–3 s) efforts with strong verbal encouragement and visual feedback. Single motor unit activity was recorded during a voluntary contraction 10% of maximum. The target torque on each side was 10% of the maximum voluntary torque for that side so that a relatively constant proportion of the available motoneuron pool on each side was tested. The target torque was produced unilaterally with the contralateral limb at rest, although torque data were recorded bilaterally during wrist and elbow flexion. The target was displayed with the voluntary torque signal and projected to ensure clear visibility for all participants regardless of eyesight. The display gain was standardized so that the data occupied ~30% of the screen. Participants were instructed to contract or “pull” until the voluntary torque matched the target and then to maintain this as steadily as possible for 6 min. At the conclusion of each trial participants fully relaxed before performing two brief MVCs to assess fatigue. Gentle verbal prompts were provided as necessary to ensure that voluntary torque matched the target as closely as possible, care was taken to avoid startle responses. Participants were unaware of the single motor unit recordings and were given no feedback regarding these data. After familiarization and practice, the study began on the less-affected side (or dominant side for healthy subjects) with 1–3 repetitions before three trials were recorded on the more-affected side (or non-dominant side for healthy subjects). This enabled participants to become familiar with the protocol on the better performing side. Trials were separated by a rest of 5–10 min to minimize the potential for fatigue. The functional ability of the patient cohorts was tested as 15 m walking speed for series 1 and with the upper-limb motor Fugl-Meyer Assessment for series 2–3.

### Data analysis

**Figure 2:**
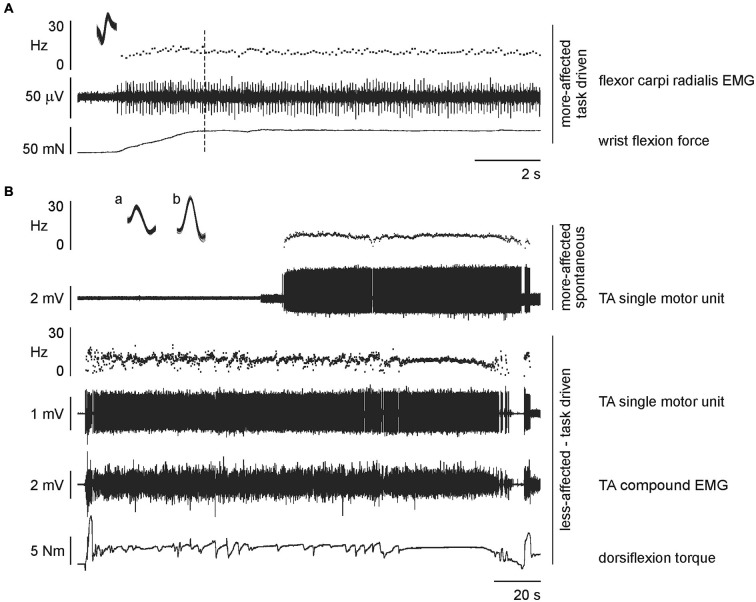
**Raw data from single stroke patients. (A)** expanded time scale recording to show a task-driven motor unit recorded on the more-affected side during wrist flexion by a 74 year old male, 11 months post-stroke. The unit was recruited at the beginning of the task (and continued to fire throughout the task) but data were analyzed from the dotted line, i.e., after the acceleration in discharge rate associated with increasing force (discharge rate 9.44 ± 0.06 Hz, mean and standard error of the mean (SEM)). Inset: superimposed action potentials demonstrating a unitary recording. **(B)** concurrently recorded units during dorsiflexion of the less-affected tibialis anterior by a 72 year old male, 39 months post-stroke. The single motor unit on the active less-affected side is task-driven with activation relating to the total torque output (discharge rate 9.55 ± 0.05 Hz, mean and SEM; superimposed spikes marked a). The single motor unit on the passive more-affected side is spontaneously active and unrelated to the task on the contralateral leg (discharge rate 8.85 ± 0.03 Hz, mean and SEM; superimposed spikes marked b).

All EMG recordings were inspected for single motor unit action potentials which were discriminated based on spike amplitude and morphology using the template matching algorithms of Spike2 software (CED, UK) during either task-driven or spontaneous activity. The activity of single motor units was analyzed from periods of stable firing so that the initial increase in firing rate following recruitment was excluded, as was any slowing prior to derecruitment. The mode of activation was determined for each single motor unit, discriminating between activity that was task-driven or spontaneously active. The former was identified by recruitment that coincided with an experimental event, and the latter when no such trigger could be identified (Figure 2). The mean firing rate was calculated over the duration of each unit’s stable discharge. Histograms were constructed for the mean interspike interval of motor unit discharge rates with a bin width of 10 Hz to enable comparison with previous reports. The dynamic range was defined as the frequency between the lowest and highest mean motor unit firing rate for a given side and series.

There were no significant differences in the firing rate or coefficient of variation of the mean firing rate for each unit between motor units on the dominant and non-dominant sides of healthy subjects when all series were combined and within each series. For this reason, and to account for the unbalanced sample between sides for the healthy subjects, these data were combined and hereafter referred to as control data. Differences in single motor unit discharge properties were investigated using a general linear model with *post hoc* Holm-Sidak pairwise comparisons. In separate analyses the dependent variable was either motor unit firing rate or the coefficient of variation of the firing rate, with factors of *series* (ankle dorsiflexion, wrist flexion) and *side* (stroke more-affected, stroke less-affected, control data). The same analyses were used to compare the firing rate and coefficient of variation of units that were task-driven or spontaneously active. The relationship between the functional ability of stroke patients, the time post-stroke and the number of motor units recorded was investigated using Pearson correlations when all series were combined and for each series. Series 3 data were not included in these statistical analyses due to the absence of control data, but were subsequently analyzed to compare the more-affected side to the less-affected side using Mann Whitney rank sum tests with Bonferroni corrections for multiple comparisons. Unless indicated otherwise, data are presented as mean ± standard error of the mean (SEM). Differences were considered significant when *p* < 0.05.

## Results

**Figure 3:**
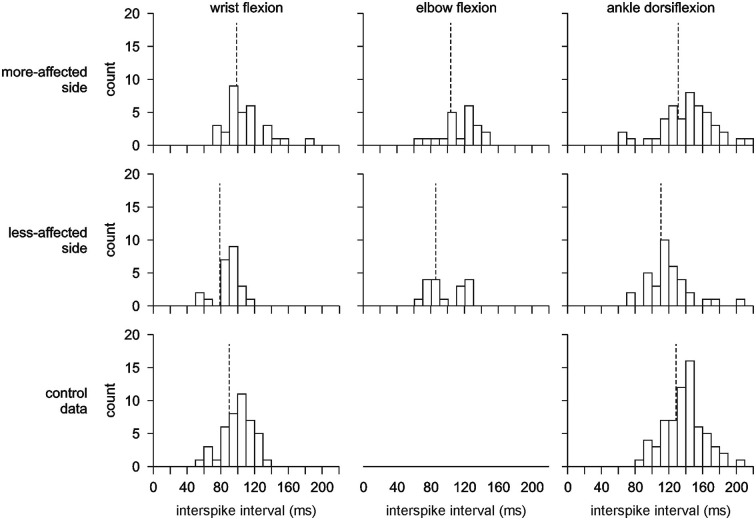
**Interspike interval histograms showing the mean discharge pattern for all motor units recorded.** There is a clear shift to the left (higher motor unit firing rates) for motor units recorded on the less-affected side compared to the more-affected side and control data. The vertical dash line indicates the mean for each histogram. Note that no control data was recorded for elbow flexion.

The number of motor units recorded in each series and each side for both stroke patients and healthy subjects are summarized in Table 1. In each experiment either three or four EMG channels were recorded on each side. In 16 instances two motor units were discriminated from the same EMG channel and in two instances three motor units were discriminated using template matching. For the remaining recordings, only one motor unit was discriminated per recording. For stroke patients there was no relationship between the level of functional ability, time post-stroke and the number of motor units recorded either when all data were combined or within each series. Single motor unit recordings were obtained for all healthy subjects in each series and for all stroke patients in the ankle dorsiflexion series, 6 of the 7 patients during wrist flexion, and 8 of 11 patients during elbow flexion. An example of raw data showing simultaneously recorded task-driven and spontaneously-active single motor unit activity is shown in Figure 2. There were no differences in the mean firing rate or coefficient of variation for motor units that were task-driven or spontaneously active, either when all series were combined or within each series. Therefore the mode of motor unit activation was not considered further. The pattern of motor unit firing during each series and on each side is presented in histograms in Figure 3. The post-trial MVCs were not different to those performed at the beginning of the study suggesting that physiological fatigue did not influence motor unit discharge properties.

### Firing rate during ankle dorsiflexion and wrist flexion

**Figure 4:**
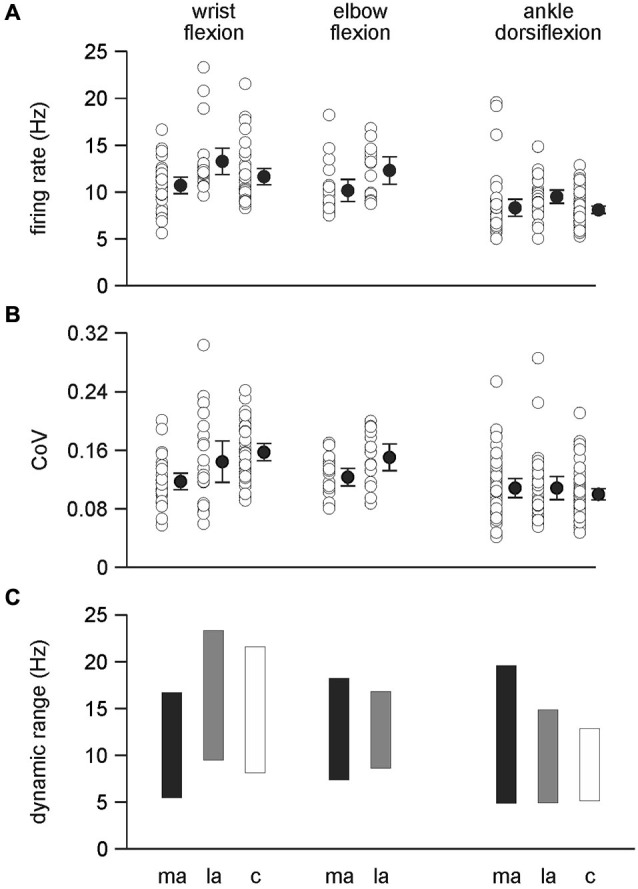
**Single motor unit discharge behavior. (A)** Firing rates were significantly higher during wrist flexion than ankle dorsiflexion (*p* < 0 .001) and also higher on the less-affected side compared to both the more-affected side and control data (*p* < 0.001). There was no difference between the more-affected side and control data for wrist flexion and ankle dorsiflexion. Firing rates were higher on the less-affected side compared to the more-affected side during elbow flexion (*p* = 0.021). **(B)** The co-efficient of variation of motor unit discharge rates was higher for wrist flexion data than ankle dorsiflexion data (*p* < 0.001). During wrist flexion the co-efficient of variation was lowest on the more-affected side compared to the less-affected side (*p* = 0.02) and control data (*p* < 0.001). There was no difference between the less-affected side and control data during wrist flexion, or between sides during ankle dorsiflexion. The co-efficient of variation during elbow flexion was lower on the more-affected side compared to the less-affected side (*p* = 0.024). **A** and **B**: data are presented for each motor unit (open symbols); mean (filled symbols) and 95% confidence intervals. **(C)** the dynamic range of mean motor unit firing rates. The dynamic range was compressed on the more-affected side only for motor units recorded during wrist flexion. ma: more-affected side; la: less-affected side; c: control data (healthy subjects).

There was a significant difference between the firing rates for single motor units during wrist flexion and ankle dorsiflexion with firing rates higher for wrist flexion 11.85 ± 0.26 Hz than for ankle dorsiflexion 8.61 ± 0.21 Hz (*F*_(1,238)_ = 93.298, *p* < 0.001) (Figures 3, 4A). Mean firing rates were highest on the less-affected side and lowest on the more-affected side of stroke patients with a significant effect of side (*F*_(2,238)_ = 9.977, *p* < 0.001). *Post hoc* pairwise comparisons revealed differences between the more- and less-affected side of stroke patients and between the less-affected side of stroke patients and control data (both *p* < 0.001), but not between the more-affected side of stroke patients and control data. Firing rates were higher on the less-affected side in each comparison. There was no interaction between side and series. The shift to higher firing rates (shorter interspike intervals) on the less-affected side after stroke is clearly evident in the histograms in Figure 3.

### Firing rate variability (coefficient of variation) during ankle dorsiflexion and wrist flexion

An interaction was found between *side* and *series* for the coefficient of variation of single motor unit discharge (*F*_(2,238)_ = 7.604, *p* < 0.001) (Figure 4B). There was a conditional effect of *series* (*F*_(1, 238)_ = 37.862, *p* < 0.001) with firing rate variability higher for combined wrist flexion data than for ankle dorsiflexion data.* Post hoc* pairwise comparisons for wrist flexion show that variability was lowest on the more-affected side compared to either the less-affected side (*p* = 0.02) or control data (*p* < 0.001), and that there were no differences between the less-affected side and control data. There were no differences in firing rate variability between sides during ankle dorsiflexion. For the conditional effect of *side* (both ankle dorsiflexion and wrist flexion series combined) (*F*_(2, 238)_ = 3.234, *p* = 0.041), *post hoc* comparisons confirmed that the firing rate variability was lower on the more-affected side compared to control data (*p* = 0.01) but that there were no differences between the more- and less-affected sides, or the less-affected side and controls.

### Motor unit behavior during elbow flexion

The firing rate of motor units during elbow flexion was higher on the less-affected side than the more-affected side (*p* = 0.021, Figures 3, 4A) as was the coefficient of variation of the firing rate (*p* = 0.024). The values for both measures fell between that of motor units recorded during ankle dorsiflexion and wrist flexion. The incidence of 4.8 motor units per subject recorded during elbow flexion was lower than that during ankle dorsiflexion, wrist flexion, or for controls (Table 1). The mean firing rate of motor units recorded during elbow flexion was not different to that recorded during wrist flexion or ankle dorsiflexion (which were different, see above).

### Dynamic range of mean motor unit firing rates for the three series

The dynamic range (the difference between the highest and lowest mean motor unit firing rate) during wrist flexion was lower on the more-affected side than the less-affected side and control data but the amplitude of the range was compressed only slightly, being 11.1, 13.7, 13.3 Hz, respectively (Figure 4C). For ankle dorsiflexion the dynamic range was not compressed on the more-affected side, being larger than both the less-affected side and control data. The magnitude of the range was 14.6 Hz for the more-affected side, 9.8 Hz for the less-affected side, and 7.6 Hz for control data. The magnitude of the dynamic range for elbow flexion was larger on the more-affected side than on the less-affected side, being 10.7 and 8.1 Hz, respectively.

## Discussion

We investigated the discharge properties of single motor units after stroke using a consistent protocol of stable low-level isometric contractions at three joints in comparison to age- and sex-matched healthy subjects. Participants were unaware of the single motor unit recordings and were instructed to hold a voluntary contraction as steadily as possible at a functionally relevant intensity 10% of maximum for a functionally appropriate duration of 6 min. The most important finding of this study was that although motor units on the more-affected side discharged at lower firing rates than those on the less-affected side as reported previously, the significant difference was that motor units on the less-affected side had a higher firing rate than those on either the less-affected side or for control data, so that the firing rate on the more-affected side was no different to control data. Firing rate variability was depressed after stroke but this was only significant for motor units on the more-affected side during wrist flexion. Similarly, the dynamic range of stable motor unit firing was compressed only on the more-affected side during wrist flexion. In contrast the dynamic range was largest on the more-affected side during ankle dorsiflexion and elbow flexion. These data suggest that motor unit discharge properties from muscles acting across a single joint after stroke cannot be generalized to muscles acting across other joints, even within the same limb. More importantly, the less-affected side does not provide a valid control for motor unit activity on the more-affected side, control data must also be recorded in age- and sex-matched healthy subjects.

### Why is single motor unit firing rate higher on the less-affected side after stroke?

The most parsimonious explanation for changes in motor unit properties after stroke is of reduced corticofugal output from the lesioned hemisphere (e.g., Cicinelli et al., 1997; Murase et al., 2004) or a reduction in the connectivity of this pathway (Stinear et al., 2007). This accords with the reduced variability of motor unit discharge in wrist muscles but does not explain increased firing rate on the less-affected side. Higher firing rates on the less-affected side may result from compensation for learned non-use of the more-affected side (Taub et al., 2006). However this explanation is unlikely because the mean MVC on the less-affected side, although higher than the more-affected side, was less than for control data. The influence of ipsilateral projections from the contralesional hemisphere is also unlikely to influence motor unit properties after stroke. In macaque monkeys this pathway was found to make little contribution to the recovery of motor function after experimental stroke (Zaaimi et al., 2012). We hypothesize that the higher firing rates on the less-affected side are due to increased excitability of the contralesional hemisphere as a consequence of reduced transcallosal inhibition from the lesioned hemisphere (Murase et al., 2004; Duque et al., 2005; Takeuchi et al., 2008; Nowak et al., 2009; Stinear et al., 2014). The resulting asymmetry in interhemispheric inhibition with cortical excitability decreased ipsilesionally and increased contralesionally, compounds the already diminished neuronal drive of the lesioned motor cortex (Murase et al., 2004), further reducing motor output and limiting functional movement and the potential for motor recovery (Duque et al., 2005; Bolognini et al., 2009; Calautti et al., 2010) on the more-affected side. It is clear however, that these changes cannot be attributed to changes in the peripheral motor axons. Changes in the biophysical properties of motor axons after stroke are subtle and because they are mostly associated with activity-dependent hyperpolarization (Jankelowitz et al., 2007), should only become apparent with fatigue, which was not evident in the current study.

### Differences in firing rate variability

The natural variabilty of motor unit discharge has been estimated as ≤40% (Gandevia et al., 1990) which may reflect a functional balance between potentiation and fatigue during sustained activity (McNulty and Macefield, 2005). In this study the variability of motor unit firing rates was not different between spontaneously-active and task-driven motor units and was lower on the more-affected side but this was only significant during wrist flexion. The origin of motor unit activity might explain some of the differences in discharge variability. One source of spontaneous motor unit activity is persistent inward currents which produced extremely low discharge variability when manifest in humans after spinal cord injury as spasm (Gorassini et al., 2004). Mottram et al. (2009, 2010) investigated whether this monoaminergic modulation of motor unit behavior might explain the origin of spontaneous motor unit discharges in spastic muscles after stroke and whether less variable discharges resulted (2010). They suggested that the discharge variability, which was ~3 times higher after stroke than after spinal cord injury, was not explained by persistent inward currents. The level of variability in the current study was similar but ~3% lower to that reported by Mottram et al. (2010) which in addition to our finding of no difference between task-driven and spontaneously active motor unit firing properties supports this suggestion. Mottram et al. (2010) proposed that motor unit discharge variability arises from tonic depolarizing synaptic drive with either a descending or segmental origin. Spontaneous activity was shown to be unrelated to spasticity, strength or force variability (Chang et al., 2013). Regardless of the mechanism, the reduced co-efficient of variation during wrist flexion presents a loss of fine motor control. This damped dynamic response may not provide the capacity for subtle responses to exogenous perturbations during functional tasks, but whether this is due to reduced neural drive or hyperexcitability at the monosynaptic Ia afferent-α-motoneuron synapse (as suggested by hyperreflexia) requires further study.

### The pattern of altered motor unit behavior

Despite different functional roles, the pattern of changes was consistent across the different series of this study although not all were statistically significant. The differences were greater in the upper limb and this may reflect the observation that the upper extremity recovers less after stroke than the lower limb and this has a larger impact on functional disability (Kalra et al., 1993; Feys et al., 1998). The changes in motor unit behavior in both the upper and lower limbs may result from altered physiology such as decreased central drive (e.g., Nielsen et al., 2008; Klein et al., 2013) but also from altered anatomy. Within muscles there may be a reduction in the number of functioning motor units (Hara et al., 2000, 2004; Arasaki et al., 2006; Lukács et al., 2008); and muscle fiber atrophy (Scelsi et al., 1984; Slager et al., 1985) associated with muscle disuse (Ramsay et al., 2011). Perhaps the least understood change in muscles after stroke, and one that will affect the behavior of individual motor units, is the pattern of denervation and reinnervation that is thought to underpin changes in muscle phenotype (for review see Hafer-Macko et al., 2008). The results of muscle biopsy after stroke are variable with both hypertrophy and increased numbers of type I muscle fibers demonstrated on the more-affected side (e.g., Edström, 1970; Dietz et al., 1986; Dattola et al., 1993; Lukács et al., 2008). Conversely, a shift from type I to type IIx muscle fibers has also been demonstrated (e.g., Frontera et al., 1997; De Deyne et al., 2004; McKenzie et al., 2009). Changes in motor unit twitch contraction times on the more-affected side (McComas et al., 1973; Young and Mayer, 1982; Dattola et al., 1993; Frontera et al., 1997) and motor unit twitch force (McComas et al., 1973; Young and Mayer, 1982; Lukács, 2005; Lukács et al., 2009) may reflect transsynaptic degeneration (McComas et al., 1973; Lukács, 2005; Lukács et al., 2009), collateral sprouting and re-innervation (Dattola et al., 1993; Kallenberg and Hermens, 2011) or a combination of these processes. The data from this study suggest that the pattern, if not the magnitude, of these changes is consistent across the more-affected side.

### Methodological considerations

Motor units on the more-affected side of stroke patients are reported to have lower peak firing rates, reduced discharge variability and a compressed firing range (Rosenfalck and Andreassen, 1980; Dietz et al., 1986; Jakobsson et al., 1992; Gemperline et al., 1995; Frontera et al., 1997; Suresh et al., 2008, 2012; Hu et al., 2012; Chou et al., 2013). Our results suggest the pattern is more complex and this may be due to methodological differences. We know of no other study to investigate the firing rate of single motor units on both sides of stroke patients in comparison to healthy subjects, except during fatigue (Hu et al., 2006). The more-affected side is most commonly compared to the less-affected side (Dietz et al., 1986; Gemperline et al., 1995; Frontera et al., 1997; Suresh et al., 2008, 2012; Hu et al., 2012; Chou et al., 2013), but occasionally the comparison is with data recorded in healthy subjects (Rosenfalck and Andreassen, 1980; Jakobsson et al., 1992). It is now beyond dispute that the side ipsilateral to the lesion is not unaffected or non-paretic, hence we use the term less-affected for this side (see Colebatch and Gandevia, 1989; Horstman et al., 2008). Our data suggest that it is only when motor unit activity on the more-affected side is compared to that of both the less-affected side and control data that the pattern of single motor unit behavior on the more-affected side after stroke can be fully understood. The motor unit discharge rates during wrist flexion were no higher than those during ankle dorsiflexion than would be expected for muscles of the upper and lower limbs (Petajan and Philip, 1969; de Luca, 1985).

The motor unit activity in this study was recorded during stable, low-level isometric voluntary contractions. The 10% MVC target was chosen to reflect the level of muscle activation typically required during everyday tasks (Thomas et al., 2005; Tikkanen et al., 2013). The target was set in proportion to the maximal output of the active side, rather than as the same absolute torque for both sides (Gemperline et al., 1995; Suresh et al., 2011). In this manner a relatively constant proportion of the available motoneurone pool was activated during all trials even when the MVC torque was asymmetric. Stable motor unit activity has previously been studied for between 2 s (e.g., Hu et al., 2012) and a maximum of 20 s (e.g., Rosenfalck and Andreassen, 1980) generally as the hold component of a trapezoidal (e.g., Chou et al., 2013) or triangular (e.g., Gemperline et al., 1995) ramp. Again, the longer duration was chosen to reflect activities of daily living such as carrying items like shopping or a dinner tray. Patients reported that the stable hold required less concentration than contractions involving ramps and changing force (unpublished data) and the consistent amplitude of the MVC at the conclusion of each trial demonstrates that physiological fatigue did not affect motor unit behavior. Finally, the motor units in this study were all recorded using surface electrodes, rather than intramuscular needle or fine wire electrodes (Rosenfalck and Andreassen, 1980; Dietz et al., 1986; Jakobsson et al., 1992; Gemperline et al., 1995; Frontera et al., 1997; Chou et al., 2013). More recent studies have decomposed single motor unit action potentials from surface array electrodes with multiple recording surfaces (Suresh et al., 2008, 2012; Hu et al., 2012). We used surface electrodes optimized for single motor unit recordings and this minimized the need for spike sorting or signal decomposition. However, the use of surface electrodes produces a bias towards more superficial motor units which in turn contain a higher proportion of fast type motor units (Čebašek et al., 1996; Kernell, 1998; Knight and Kamen, 2005) and this bias may in part, explain some of the differences found in this study.

A large proportion of motor units recorded in this study were active on the contralateral “resting” side. Although we could not simultaneously record torque contralaterally during ankle dorsiflexion contractions, torque was recorded bilaterally during wrist and elbow flexion contractions to ensure the posture of the resting limb was the same on both sides and that there were no changes in passive tension. There was no discernible torque recorded that could be associated with the activity of motor units on the contralateral side. There are two possible explanations for the absence of recorded torque. First, although the force transducers used in this study have a linear response they are not designed to record the torque produced by single motor units. Second, the first recruited motor units of a voluntary contraction (McNulty and Cresswell, 2004) or a single active motor unit in an otherwise quiescent muscle (McNulty and Macefield, 2005) may not generate measureable force as their action is thought to increase internal muscle tension and stiffness so that the slack in the muscle-tendon unit is reduced, allowing any subsequent increase in tension to be transduced. The activation of contralateral motor units could reflect bilateral projections to skeletal muscles (Colebatch et al., 1990; Ridderikhoff et al., 2005) or contralateral irradiation (Zijdewind and Kernell, 2001) but presumably only for task-driven motor units. The majority of contralateral motor units were spontaneously active and as such their discharge is unlikely to be related to the descending motor command. The origin of this activity remains uncertain.

### Limitations

The SMU electrodes used in this study were designed for an amplifier using a low-pass filter of 450 Hz, half that commonly used for surface EMG recordings. However this did not affect either the identification or the discrimination of single motor unit potentials in this study for several reasons. First, the power of the EMG signal lies below 100 Hz; second, because the majority of units were spontaneously active, their action potentials were not superimposed on compound EMG; and finally, there was no difference in the ability to discriminate units recorded using these electrodes and those recorded using standard surface electrodes and a 1,000 Hz low-pass filter.

In this study the behavior of motor units recorded during elbow flexion fell between that recorded during ankle dorsiflexion and wrist flexion. These data were recorded in stroke subjects only (there being no effect of side in healthy subjects at the wrist) to exclude the potential confounding effect of handedness, particularly if the more-affected hand had been dominant pre-stroke. The effect of handedness should be more pronounced in distal compared to proximal muscles of the upper-limb. The absence of control data during elbow flexion is a limitation of the study. Evidence for the effect of hand dominance or lesion laterality on recovery after stroke is not definitive. A dominant more-affected hand resulted in less impairment than a non-dominant more-affected hand, although this difference was only apparent in functional assessments and not in the performance of activities of daily living (Harris and Eng, 2006). A strong hand preference in monkeys, regardless of side, was associated with less recovery than more bilateral hand use and this was independent of lesion volume (Darling et al., 2013). Handedness influenced the extent of less-affected hand use but not that of the more-affected hand in stroke survivors (Rinehart et al., 2009), and there was no effect of handedness for Constraint-induced Movement Therapy in which the less-affected hand is restrained (Langan and van Donkelaar, 2008). That motor unit recordings from biceps brachii followed the pattern of the wrist flexion suggests that our results during wrist flexion are not affected by handedness. The difference in the properties of motor units of muscles acting on the wrist and elbow can be explained by a lower innervation ratio (Buchthal and Schmalbruch, 1980), more numerous monosynaptic corticospinal projections (Palmer and Ashby, 1992), and fewer bilateral projections in distal muscles such as those controlling the wrist compared to more proximal muscles flexing the elbow (Colebatch et al., 1990).

### Conclusions

This study examined the discharge properties of single motor units during contractions acting on joints in the upper and lower limbs of both sides after stroke and in healthy age- and sex-matched controls. The pattern of changes, but not the magnitude, was consistent across the more-affected side after stroke. Although motor units on the more-affected side had lower firing rates compared to the less-affected side as reported previously, this difference does not reveal the true pattern of altered motor unit behavior after stroke. The most significant finding of this study was that motor unit firing rates on the more-affected side were not different to control data. Rather the pathological change in motor unit firing rate occurred on the less-affected side, where mean rates were significantly higher than either the more-affected side or control data. Motor unit discharge variability was lower, but this was only significant for wrist muscles on the more-affected side. We hypothesize both changes reflect the asymmetric interhemispheric inhibition known to develop after stroke. Most importantly, this study demonstrates that in order to understand physiological changes after stroke it is necessary to compare data from the more-affected side, the less-affected side *and* healthy age-and sex-matched subjects.

## Author contributions

Penelope A. McNulty Conceived and designed the experiments, supervised data collection and analysis, wrote and revised the manuscript, approved the final version.

Gaven G. Lin Performed data acquisition and analysis, contributed to manuscript drafting and revision, approved the final version.

Catherine G. Doust Performed data collection and analysis, contributed to manuscript drafting and revision, approved the final version.

## Conflict of interest statement

The authors declare that the research was conducted in the absence of any commercial or financial relationships that could be construed as a potential conflict of interest.
